# Challenging dominant breast cancer research agendas: perspectives on the outcomes of the interagency breast cancer and environment research coordinating committee

**DOI:** 10.1186/s12940-019-0479-1

**Published:** 2019-05-06

**Authors:** Lauren Richter

**Affiliations:** 1 Social Science Environmental Health Research Institute, Northeastern University, Boston, 360 Huntington Avenue, 318 INV, Boston, Massachusetts 02115 USA; 2 Silent Spring Institute, 320 Nevada Street, Suite 302, Newton, MA 02460 USA

**Keywords:** Breast Cancer, Cancer Prevention, Environmental Contributors to Cancer, Social Movements

## Abstract

**Background:**

As breast cancer rates increase globally, there is growing scientific consensus that greater understanding of the causes of breast cancer is needed to better prevent its occurrence. Genetics accounts for a small percentage of cases, thus environmental factors and epigenetics are increasingly suspect in breast cancer etiology. Within the breast cancer and environmental breast cancer social movements, there are longstanding calls for research and policy aimed toward the prevention of breast cancer. To better understand the opportunities and barriers to addressing environmental contributors to breast cancer, this article investigates both outcomes and perceptions of stakeholders involved in the Interagency Breast Cancer and Environment Research Coordinating Committee (IBCERCC). The IBCERCC was mandated by the 2008 U.S. Breast Cancer and Environmental Research Act, a law representing years of advocate and researcher efforts to produce national strategies and federal funding for breast cancer prevention research.

**Methods:**

To understand the meaning and impact of the IBCERCC advisory committee and final report, *Prioritizing Prevention*, I draw on fifteen confidential semi-structured interviews with members of the twenty-five person IBCERCC, in addition to six confidential semi-structured interviews with key breast cancer funders, advocates, and researchers affiliated with national reports on environmental contributors to cancer. I examine media coverage, congressional hearing transcripts, and official responses to the release of the IBCERCC report by governmental and non-governmental organizations.

**Results:**

Interviews and publicly available documents reveal a set of direct and indirect outcomes of the 2013 IBCERCC report. Interviewees in government positions perceived the 2014 renewal of the Breast Cancer and the Environment Research Program to result from IBCERCC efforts, notable in the context of declining U.S. federal research funding. Interviews also revealed a suite of potential barriers to the implementation of report recommendations including: distinct interpretations of the federal mandate, disparate assessments of scientific evidence, government funding crises, and lack of specificity around responsibility for implementation of report findings.

**Conclusion:**

This article examines efforts to shift institutional research and funding priorities in cancer research towards prevention. Social science research can support efforts to shift institutional priorities by identifying broader social contexts and underlying values typically unnamed in scientific discourse

**Electronic supplementary material:**

The online version of this article (10.1186/s12940-019-0479-1) contains supplementary material, which is available to authorized users.

## Background

The lifetime risk of breast cancer in the U.S. has increased from 1 in 22 in the 1940s to 1 in 8 today, and is the most common cancer in women, with most cases occurring in individuals with no family history [1]. A scientist on the Interagency Breast Cancer and Environment Research Coordinating Committee (IBCERCC) estimated that 20-30% of the causes of breast cancer are understood, concluding that we remain largely in the dark about what causes this disease. Though progress has been made extending the lives of disease sufferers, U.S. research funding has not substantially addressed cancer prevention and specifically research on the environmental causes of the disease. Environment in this context is broadly understood as: *non-voluntary* (exposures individuals cannot fully control, or may involuntarily experience through activities like cosmetic use), or *voluntary* (exposures linked to lifestyle choices) [1–4].

Disease sufferers, advocates, and researchers are concerned with the prevalence of breast cancer and disproportionate allocation of research funding for cancer treatment over prevention [4–8]. Social science scholars have demonstrated the strong influence of the breast cancer and environmental breast cancer social movements in shaping federal, private, and NGO research funding on breast cancer treatment and prevention [5, 9, 10]. Public health oriented stakeholders have built momentum for improved scientific models and policy recommendations that could address breast cancer prevention, highlighted in a series of major reports such as the 2010 President’s Cancer Panel (PCP) report, the 2012 Institute of Medicine (IOM) report, and the 2013 Interagency Breast Cancer and Environmental Research Coordinating Committee (hereafter IBCERCC). This paper situates the IBCERCC’s *Prioritizing Prevention* report in the larger trajectory of the environmental breast cancer movement. The 2013 IBCERCC report is the outcome of the 2008 Breast Cancer and Environmental Research Act, which itself is an outcome of national organizing and strategies developed in the 1990s by breast cancer advocates. This article seeks to evaluate the outcomes of the IBCERCC, while contextualizing this committee and report as longer-term outcomes of multi-decade breast cancer advocacy and grassroots organizing. I assess participant and stakeholder opinions on the impetus for the formation of the IBCERCC, the state of evidence on environmental causes of breast cancer and the implications of the IBCERCC efforts and final report. This research is based on in-depth semi-structured interviews with members of the twenty-five person IBCERCC, in addition to breast cancer stakeholders affiliated with national breast cancer research funders, advocacy organizations, and researchers involved in the 2010 President’s Cancer Panel report and 2012 Institute of Medicine report. All interviews were confidential, and efforts have been made to protect the identities and affiliations of interviewees [11]. Additional data come from Congressional hearing transcripts, media coverage of the IBCERCC report, and written responses from the Department of Health and Human Services regarding the implementation of IBCERCC recommendations.

## Methods

This article investigates the impact of the IBCERCC report from the perspective of committee participants and engaged stakeholders, in order to answer the question: what have been the outcomes of the IBCERCC effort and report, if any? Each member of the IBCERCC committee was directly approached (including the executive secretaries) by the researcher, and fifteen semi-structured phone and in-person interviews were completed between March and July of 2014. Of the total twenty-five IBCERCC members, five members declined to participate and five additional members did not respond. As mandated by the 2008 law, the committee was comprised of federal employees and non-federal representatives employed as scientists, physicians, and advocates. Interviews solicited information about members’ experiences on the IBCERCC panel, opinions on the 2008 law and 2013 final report, and awareness of outcomes. Participants were asked questions pertaining to the nature of their involvement on IBCERCC subcommittees, initial expectations, perceived personal impact on the committee, overall impression of the report, and what individual, organizational or other changes they have made or know of since the report’s release in February of 2013. Over the course of data collection, I expanded the dataset through snowball sampling to include six additional stakeholders in breast cancer funding, research, and advocacy, such as contributors to other major federal reports on environmental contributors to cancer. I include interviewee discussion of the 2010 PCP and 2012 IOM reports as they were discussed as indicators of a growing “zeitgeist” around environmental contributors to cancer across the life course. All interviews were recorded, transcribed, and de-identified, except for one interviewee who wished not to be recorded.

Additional data came from online research using LexisNexis Academic media to capture newspaper and blog coverage of the IBCERCC report’s release, and searches of websites of major breast cancer organizations to locate statements and press releases about the report. Proquest Congressional provided data on the Breast Cancer and Environment Research Act, including testimony of pertinent congressional hearings and prior versions of bill language. Finally, the Collaborative on Health and Environment (CHE) webinar “Four Years After the President’s Cancer Panel Report: Recommendations and Next Steps” on 10/20/14 featured presentations from one IBCERCC member and one academic advisor to the 2010 PCP.

In order to assess the outcomes of the report, qualitative interviews were conducted using a semi-structured interview approach, allowing for follow-up questions and open-ended input from interviewees. Interviews served as the primary research method in order to analyze interpretations of outcomes, decision-making processes pertinent to stakeholder action since the report’s release, and knowledge of organizational shifts. Interviews were transcribed and uploaded into the qualitative analysis program Dedoose. Topical codes were developed based on the research questions, and thematic codes were developed as patterns emerged in line-by-line analysis of interview transcripts [12]. For example, it became clear that stakeholders adhered to different definitions of “environment” in the context of exposure (i.e. using environment to refer to lifestyle or so-called “voluntary” exposures vs. “non-voluntary” exposures such as those related to pollution or specific chemicals). Distinct definitions of environment as it pertains to cancer causes substantially impact assessments of the adequacy and implementation of the IBCERCC and other cancer reports, echoing findings by Baralt and McCormick (2010) and Klawiter (2008).

While there are a range of approaches to evaluate social movements and public policy outcomes, this article uses Guston’s approach to evaluating the public or achievement-oriented impacts of organized scientific consensus [13]. Guston distinguishes between direct impacts or “a change in any authoritative public decision, including changes in legislation, funding, regulations, or other concrete consequences,” and indirect impacts, considering both substantive change and procedural changes that may not appear as quickly and may (or may not) expand over time [13]. In addition to drawing on Guston’s framework, I identify and discuss potential barriers to the implementation of report recommendations, highlighting implicit stakeholder assumptions revealed in the data.

### Breast Cancer Prevention in Context

Public knowledge of and funding for breast cancer research can be attributed to decades of outcry by disease sufferers, direct actions (letter writing, signature gathering, protests), grassroots organizing, federal research funding, and contemporary professionalized non-governmental organization advocacy, and corporate research fundraising. Research on breast cancer movement(s) in the U.S. traces shifts in movement strategy, tactics, and political opportunity from the early 1970s to the present day. Prior to the mid-1970s, there was longstanding concern with cancer in the U.S., as evidenced by the establishment of the National Cancer Institute (NCI) in 1937, the establishment of the Section on Environmental Cancer within the NCI in 1948, and the 1971 National Cancer Act. Notably, the NCI Section on Environmental Cancer was closed in 1964, and the head of this section, Dr. Willhem Hueper, criticized trends in American cancer research away from case study research focused on occupational exposure and towards large-scale epidemiological studies of so-called lifestyle factors, like smoking [14, 15]. Hueper’s concerns regarding the overall shift of cancer research agendas towards almost exclusively individual factors have largely come to fruition [14].

Until the early 1970s, breast cancer was predominantly seen as both an individualized and stigmatized concern for women (at a time when most health research was conducted on white men). This changed with the “personal is political” cultural shift due to progressive movements including the feminist movement [5, 10, 16]. The breast cancer movement that emerged in the 1970s and 1980s focused on improving treatment options, informed consent and detection methods, with increasing attention towards lobbying as a strategy to improve treatment options and access to mammography [16]. By the 1990s the movement continued to press for access to treatment, but some activists shifted focus to increasing explicit federal government funding for breast cancer research. The movement shifted frames in the 1990s from an individual diagnosis and treatment focus to a public health crisis frame, thus requiring a coordinated federal response [16, 17]. At this time, inadequate research funding was framed as institutional neglect of a public health crisis disproportionately impacting women.

Professionalized national breast cancer organizations emerged in the 1990s, distinct from the more dispersed grassroots movements comprised of local volunteer-based groups. One significant organization was the National Breast Cancer Coalition (NBCC), expanding rapidly from eight to 300 member organizations between 1991 and 1994 [7, 16], with 600 member organizations in 2014. The NBCC has been successful by many accounts, securing significant disease-specific policymaking and investment in research (approximately $3 billion total new funds specifically for breast cancer research according to NBCC’s website). While the growth in federal, nonprofit, and private sector research on breast cancer has increased dramatically, advocacy groups and scientists have long criticized cancer research for neglecting to adequately examine non-voluntary environmental contributors [6, 7, 18–20].

#### The Environmental Breast Cancer Movement

An environmental breast cancer movement (EBCM) emerged from the breast cancer and environmental movements, with specific concern over the health effects of toxicants, endocrine disruptors, and failure of mainstream biomedical research models to address the causation of cancer [5]. Brown situates his analysis of the environmental breast cancer movement in a larger theoretical framework resting on the tension between what he calls the “dominant epidemiological paradigm” (DEP) and the alternative “public paradigm” proposed by environmental health activists. Drawing on Kuhn, he writes:

a paradigm refers to an established worldview that shapes what problems scientists are encouraged to study and how to study them, but excludes those theories, hypotheses, and observations that do not match this worldview. Major alterations in science occur when a critical mass of researchers begin to question the validity of a dominant paradigm that no longer adequately explains empirical evidence [5].

In the case of the breast cancer movement, there are tensions between the investigators and institutions comprising the DEP (which generally focus on individual, behavioral, and genetic contributors to illness), and advocates, scientists (and some regulators) who desire stricter chemical regulation and increased research on non-voluntary environmental causes of the disease adhering to a public paradigm [21]. The NBCC mobilized significant national support and political pressure to successfully win the Department of Defense Breast Cancer Research Program, requiring advocates sit on grant review panels for research funding. In addition to other victories, grassroots non-governmental organizations (NGOs) such as the Huntington Breast Cancer Action Coalition on Long Island initiated the Long Island Breast Cancer study and helped launch the Breast Cancer and the Environment Research Program (BCERP) at the National Institute of Environmental Health Sciences (NIEHS) with support from the NCI [1, 17]. While there is increasing federal recognition of non-voluntary, chemical-based environmental contributors to breast cancer, overall research funding on breast cancer prevention (irrespective of the suspected causes, be they smoking or air pollution) is limited to less than 10% of federal breast cancer funding [1]. In 2019, breast cancer researchers and advocates learned that the BCERP program would be ending.

#### The 2008 Law and IBCERCC Report

The NBCC held two Environmental Policy Summits in the 1990s. An outcome of these summits was the pursuit of a new bill creating “a national strategy and a program to competitively fund collaborations of researchers and community groups” focused on breast cancer [22]. The bill that eventually became the 2008 Breast Cancer and Environment Research Act was first introduced in 1999. Between 1999 and 2008 the bill shifted away from NBCC’s immediate goal of funding new research. Congressional testimony reveals that members of the National Institutes of Health (NIH) were concerned with passing a bill that specifically mandated what types of research could be funded. The issue raised was that such a constraint might threaten peer-review scientific integrity [23]. By 2008, NBCC was able to secure NIH support for a modified version of the bill, however, the final bill that passed shifted from funding research to funding an assessment and report, a move that upset a number of breast cancer advocates, including NBCC.

The final Breast Cancer and Environmental Research Act of 2008 states that the law’s aim is to “reduce the burden of breast cancer on women and men of all ethnic groups”[1]. Under this U.S. law, the Secretary of Health and Human Services established the IBCERCC to accomplish the following:

- Review federal research efforts concerning the environmental and genomic factors related to breast cancer.
- Identify scientific advances in breast cancer research and outline key research questions, methodologies, and knowledge gaps.
- Develop a comprehensive strategy for accelerating transdisciplinary, innovative, and collaborative research on breast cancer and the environment across federal agencies and in partnership with nonfederal organizations.
- Determine how to increase public participation in decisions about breast cancer research and the optimal mode of dissemination of information on research progress. [1].

The 2008 law specified that the IBCERCC be “comprised of federal members from agencies involved in research on breast cancer and the environment, including the National Institute of Environmental Health Sciences (NIEHS), National Cancer Institute (NCI), Environmental Protection Agency (EPA), the Department of Defense (DOD), and the Center for Disease Control (CDC); non-federal members from scientific and clinical communities; and non-federal members who represent individuals with breast cancer”[1]. For the IBCERCC report, environment is defined broadly as:

- Lifestyle and behavioral factors, such as alcohol intake and physical activity.
- Chemical agents that people are exposed to through pesticides, industrial pollutants, consumer products, and medications.
- Physical agents, such as radiation from medical and other environmental sources and other nonchemical substances.
- Social and cultural influences, such as family, community, psychosocial/social, and societal factors that may influence breast cancer risk.

The report makes seven overarching recommendations: prioritize prevention, transform how research is conducted, intensify the study of chemical and physical factors, plan strategically across federal agencies, engage public stakeholders, train transdisciplinary researchers and translate and communicate science to society. The report also provides a comprehensive state of the science of breast cancer research, and summarizes ongoing research pertaining to breast cancer and the environment as defined by IBCERCC. Over 100 recommendations were made in the report, with over half focusing on specific research recommendations.

## Results

This section is broken into two parts: First, I describe specific direct and indirect impacts of the IBCERCC process and report as described by interviewees. Second, I identify potential barriers to implementation of report recommendations that include: discrepant conceptions of the 2008 mandate and committee intent, disparate assessments of the strength of scientific evidence, federal budget crises, and responsibility for implementation of report recommendations.

The 2013 IBCERCC was the outcome of a federal mandate to summarize extant science and federal research funding on environmental contributors to breast cancer, while simultaneously identifying areas for future research, policy, and regulatory action. Data collected reveal a number of impacts, primarily in the areas of producing and disseminating a robust state of the science and helping maintain federal funding for transdisciplinary research on environmental contributors to breast cancer (at least in the years immediately following the report’s release). When asked about the impacts of the report, interviewees revealed a number of discrepant perspectives on the purpose and outcomes of the IBCERCC, as well as the 2010 President’s Cancer Panel and the 2012 Institute of Medicine reports. As one interviewee said, if you assemble a large group of people with different training and agendas, there will be disagreements.

Social science scholarship has long identified the tensions in efforts to establish scientific consensus, noting that scientific deliberations always contain underlying assumptions and potentially conflicting frames [24]. Silvia Tesh writes that “firmly but often unconsciously held answers” to questions such as “what is the legitimate source of knowledge,” fundamentally “guide scientists, policy makers, and ordinary citizens alike to different constellations of facts about the causes of disease and, hence, to different preferences for prevention policy”[25]. I echo Tesh’s call to identify the inevitable presence of values in science and policy, and for these values and their implications to be openly identified. In addition to the topical findings on direct and indirect impacts, I found discrepant beliefs about the IBCERCC mandate, strength and implications of evidence, federal budget crises, and responsibility for implementation.

### Direct and Indirect Impacts

Interviewees were asked about their knowledge of impacts or any changes they had implemented in their research or university departments, campaign work, within their agencies or hospitals as a result of this report. Some felt there had not been any actual outcomes, or were unaware of impacts at the time of their interview. Interviewees employed by the federal government or in leadership positions on national report committees had more access to information regarding federal grant-maker response to the IBCERCC report. Some interviewees characterized direct outcomes of the report as one federal grant opportunity and one existing grant renewal. These include the release of a request for applications (RFA) 12/5/14: “Coordinating Center for the Breast Cancer and the Environment Research Program [BCERP],” and under a related funding opportunity, the NIEHS and the NCI released a renewed RFA: “Environmental Influences during Windows of Susceptibility in Breast Cancer Risk.” Many interviewees felt that this extended funding for the BCERP could, in part, be attributed to the IBCERCC report and momentum for further research on breast cancer prevention. Two interviewees pointed out that no dollar amounts were provided in the IBCERCC report for target research funding amounts, so perceptions of the adequacy of these federal RFA varied. Given that the federal IBCERCC report calls for a reallocation of current funding towards prevention-based research, the extent to which the above BCERP renewal and funding opportunities were considered substantial were mixed in 2014. One committee member said, “there was tremendous resistance to a number of us wanting to call out a percentage of investment of research dollars to be spent. That, we did not get, if you noticed.”

While some scientists felt their research agendas already fell within the scope of research the report called for, at least one academic scientist indicated that their research had shifted towards chemical contributors to breast cancer. This interviewee felt that breast cancer advocates wanted to know this information, and that this concern warranted a response. In a response to an inquiry from the NGO Breast Cancer Prevention Partners (formerly the Breast Cancer Fund) on implementation of IBCERCC recommendations, then Secretary of Department of Health and Human Services, Kathleen Sebelius, wrote: “The NTP (National Toxicology Program) and U.S. Environmental Protection Agency are attempting to develop adequate, reliable high-throughput cell-based models for the breast so that chemical screening of the thousands of untested registered chemicals on the market can be evaluated for deleterious effects.” This letter also noted that part of the NCI’s “provocative questions” initiative for new areas of funding called for research looking into biological mechanisms that influence susceptibility to cancer during different periods of the life course. Additional outcomes include a number of advocate interviewees reporting that they used report findings to inform their outreach and trainings in communities. One advocate felt the IBCERCC report helped inform NIEHS’ most recent strategic plan, an accomplishment “they could hang their hat on.” Finally, two academic researchers noted that the IBCERCC, the 2010 President’s Cancer Panel, and 2012 Institute of Medicine reports were important indicators of a change in cancer research culture from a decade ago, when research on environmental contributors to cancer was not given substantial attention and more difficult to publish in peer-reviewed journals.

Regarding potential indirect and broader impacts, interviewees reported on activities related to the report’s dissemination publicly and within agencies [13]. Committee members published a special issue in the journal *Reproductive Toxicology* focused on environmental impacts on breast development and disease [26]. In terms of media coverage, several high profile outlets covered the report’s release, including the *New York Times*, *San Francisco Chronicle,* and *Forbes*. LexisNexis media searches for all three reports indicate that the 2010 President’s Cancer Panel report received significantly more media coverage than either the 2012 Institute of Medicine or 2013 IBCERCC report. One interviewee felt that the President’s Cancer Panel report received so much attention because it was the first major federal report on this topic and covered environmental contributors to all cancers. Additionally, advocates and federal employees reported distributing the report within their professional networks. For example, Breast Cancer Prevention Partners organized presentations and webinars on the report, gave copies to all members of the California Congressional delegation, and integrated report findings into congressional testimony on the Toxic Substances Control Act (TSCA) reform hearings.

### Potential Barriers to Implementation

While some interviewees cited a number of outcomes from the report in the areas of academic and internal agency research, data raise concerns regarding potential barriers toward achieving progress in the following areas: planning strategically across federal agencies, engaging public stakeholders, and prioritizing cancer prevention in research and policy. A number of members expressed a range of concerns over why they perceived a lack of implementation of the committee’s recommendations, with reasons ranging from persistent federal budget crises to a lack of “super audience” (understood as a political champion) to oversee and help enact the report’s recommendations. At least two respondents suggested that expecting results within approximately 2 years of a report’s release (at the time of interviews) does not allow enough time for needed changes to come about within government. This perception contrasts with the views of long-term advocates who trace the roots of the 2013 IBCERCC to campaigning since the 1990s, the introduction of a bill in 1999, and the passage of the law in 2008. Of the advocates interviewed, one expressed concern with IBCERCC report’s overall framework, explaining that the focus on environmental chemical exposures was misguided. This interviewee felt that regulating chemicals will not save women quickly enough compared to a preventive vaccine. In contrast, another advocate felt that the “the problem is that the whole system is dominated by medical approaches and using science and research for economic development,” as opposed to a focus by regulators, agencies, scientist and medical practitioners on non-voluntary environmental exposures to populations that can be controlled (i.e. by substantially regulating new and existing chemical compounds such as asbestos or new replacement per-and poly-fluorinated compounds).

### Interpretations of Mandate Intent and Scope

Members from federal agencies, advocates, and cancer funders at times held distinct conceptions of the legal mandate’s intent and appropriate scope. Interviewees expressed distinct conceptions of their own versus other stakeholder group capacity for making change, especially for challenging the status quo within institutional settings. For example, one interviewee reported that one larger national NGO cancer research funder concluded that they needed to increase investment in cancer prevention. This individual was able to tell senior leadership “here is another document that calls for prevention in cancer, in this case breast cancer, and provide some direction for where we should be funding.” Despite gaps and direction provided in the IBCERCC report, this interviewee noted that this major funder’s work on prevention would focus on colorectal cancer, as some felt it was an underfunded type of cancer. In contrast, in a *Forbes* article covering the release of the IBCERCC report, Julia Brody, Executive Director of the Silent Spring Institute argued that NCI and major NGO cancer research funders could take immediate action to fill the research gaps in breast cancer prevention identified by the report [27].

Interviews revealed multi-layered understandings of both individual and institutional capacity to bring about changes called for by the IBCERCC report. While interdisciplinary, inter-agency, multi-stakeholder collaborations are heralded as necessary for tackling complex health problems, there are frequently differential distributions of prestige by affiliation and type of expertise. One individual felt that the ability of researchers to suggest shifts in federal funding (on the IBCERCC) could be constrained by perceived conflicts of interest related to their personal research interests. Many members felt that either their own or other stakeholder participation was constrained politically. For example, regarding the challenges of shifting federal research funding towards prevention, one interviewee said:

It was pretty clear that, in my view, the agency representatives were in a really tough place. They weren’t really representing their agencies … The researchers had a little bit more fluidity in their ability to talk about issues.

One federal agency participant indicated that the report’s recommendations were largely not within their agency’s capacity to implement given that their agency’s research pertinent to cancer is conducted in conjunction with other agencies. They furthermore noted that the mandate of the IBCERCC was not to conduct risk assessment or provide regulatory input.

While some felt that the IBCERCC influenced the NIEHS strategic plan and NIEHS/NCI funding quite directly, some individuals indicated that the report’s mandate and findings had little impact on their own work or institution. Another interviewee from an NGO cancer research funder said that they were already doing research pertinent to voluntary environmental causes of cancer, citing their research on tobacco smoke. One academic researcher noted that they already did research on diet and breast cancer, therefore they felt the recommendations did not shift their current work. These examples raise the issue of specifying definitions of environment and within those definitions (e.g. voluntary, non-voluntary, chemical) specifying the type of prevention (primary, secondary or tertiary) a given strategy seeks to address. Baralt and McCormick found that 95% of advocates involved with BCERP were concerned primarily with non-voluntary environmental exposures, with scientists being more concerned with a combination of both non-voluntary and voluntary exposures [4]. While the IBCERCC report explicitly assesses research on various voluntary and non-voluntary exposures and makes recommendations according to this division, this level of specificity was frequently absent from many interviews. Without this level of specificity, for example in federal cancer prevention funding databases, it is difficult to accurately track the degree to which the IBCERCC recommendations are shifting research and funding towards underfunded non-voluntary chemical exposure research (i.e. toxicants in consumer goods) or voluntary risks like food choices. It is important to add here that social scientists, public health scholars, and advocates argue that even the “voluntary” lifestyle risk factors like diet, exercise, and tobacco use have social structural components beyond individual-scale choice.

### Strength of the Science

The strength of scientific evidence is a contentious issue in the field of breast cancer prevention research, policy, and advocacy. For example, in their research on expert interpretations of biomonitoring data, Shamasunder and Morello-Frosch note the long-standing dispute between environmental health and justice movement advocates, industry, and regulators over how data on chemical exposures, hazard assessments, and cumulative effects should be integrated into chemical regulation [24]. These disputes often fall along the lines of how to evaluate data from lab-based animal studies vs. human epidemiology or exposure studies. Industry and regulators typically emphasize quantitative probability estimates of health effects, stressing, “the importance of risk-based responses that quantify the probability of individual and population harm from chemical exposures” [24]. Interviews revealed disagreement over the types and quantity of evidence pertaining to environmental causes of breast cancer. Regarding the issue of evidence and advising precautionary consumption (i.e. individuals avoiding particular ingredients or materials) or even regulation, one academic scientist said they were against recommending precaution, because they felt that while numerous compounds cause breast cancer in animals, there is not sufficient evidence that they cause it in humans. While some committee members felt that there was enough evidence to recommend that women avoid these compounds, this scientist felt that they were obligated to adhere to the so-called “gold standard”:

in which you really need to have definitive proof before you give the public any specific recommendations to avoid something. The only thing I could really say that causes breast cancer with any degree of certainty was ionizing radiation.

In contrast to the above position, nearly all the advocates and many other interviewees would echo the assessment below by Brody in *Reviews on Environmental Health*:

We must stop allowing medical research programs – which are based on human clinical trials and epidemiologic studies of exposures you can see or ask about – to impede progress in environmental health. We must stop allowing statements that there is ‘no *proof* that A causes B’ to block action based on the weight of evidence that we do have. Instead, we must build an environmental health paradigm for long-latency disease in which we rely on animal and cell studies of biological mechanisms coupled with human exposure studies, using these types of evidence as a basis for public health intervention to reduce exposure. Epidemiologic evidence is expected to lag behind, while we act judiciously on early warnings from studies that show a chemical affects cancer mechanisms in animals or cells, and people are substantially exposed [8].

One federal employee noted that some colleagues in their office who read the draft report were concerned with specific language around chemical determinants, pointing out that the IBCERCC was not intended to serve as a risk assessment document nor was it intended to inform regulations directly. The concern was also motivated by the desire to avoid alarming the public about chemical risk. While one federal employee expressed concern about the language in the report being too strong, another federal employee felt concerned that the language was toned down over subsequent drafts, resulting in sections that were too cautious in their interpretation of the science (in this case on chemical contributors to breast cancer). Notably, there was limited discussion in the interviews of the primary U.S. chemical regulatory apparatus (TSCA) explicitly, however a number of the interviewees did mention the need to better regulate chemical production.

### Budget Crises

Interviewees cited recurrent federal budget crises and the government shutdown in early 2013 as a common reason for limited attention to the report and hindered implementation of recommendations. A number of federal and scientist committee members commented that people were just trying to hold on to what funding they had, and furthermore that the current research funding climate was not conducive to starting new, innovative research initiatives. Concerning BCERP funding, one federal employee commented that: “the NIH budget has been shrinking overall, so it actually says something that the program [BCERP] size won’t shrink in the face of reduced budget opportunities.” For advocates, many of whom were engaged in work on breast cancer with little to no funding, federal budget crises did not hold as much weight as an explanation for limited action. Most respondents echoed a point made the IBCERCC report, that the breast cancer movement has been instrumental in pushing for research on this issue through a number of means. The advocates generally held a more proactive, power-oriented model of change regarding the relationship between government, science, regulation, and thus felt the need for social movements and advocates to actively build momentum for changing research agendas. Other concerns around budget were attributed to perceptions of bad timing, including the Secretary of HHS having to focus on the Affordable Care Act implementation in early 2013.

### Responsibility for Implementation

Interviewees raised a number of issues pertaining to responsibility for implementation of the committee’s recommendations. As one interviewee noted, it was not clear in the report’s mandate who or what super-audience the report had, aside from NCI and NIEHS as sponsors. This lack of clarity around who or what entity was responsible for overseeing the enactment of recommendations may relate to the mandate for this report representing a decade of policy negotiations that ultimately did not align with the requests of the advocacy organization behind the impetus for the 2008 federal law, NBCC. While it is beyond the scope of this study, it is notable that the original bill was introduced in the late 1990s. Shifting political dynamics within the U.S. congress and between sectors of the breast cancer movement(s) may have posed a challenge to securing research funding for a specific type of breast cancer prevention research. A related concern raised regarding the implementation of the report was that the report did not consistently name the specific agencies, institutions, or individuals responsible for carrying out specific recommendations. That concern was noted in contrast to implementation recommendations made in the 2010 President’s Cancer Panel, which explicitly delineate responsibilities to discrete parties. However, in an interview with an individual closely involved with this report, they noted that all President’s Cancer Panel reports list responsible parties. They did not feel that this necessarily made an impact on whether recommendations would be implemented or not.

A further concern is the question of if and how breast cancer research should relate to public policy. This came up in a number of interviews, demonstrating some hesitancy on the part of scientists, particularly those from federal agencies to make explicit policy recommendations, especially pertaining to the regulation of chemicals. Three interviewees illustrate distinct views and tensions regarding the place of policy in the IBCERCC report’s recommendations:

Federal staff: It [the final report] wasn’t designed to impact regulatory policy, policy is different from regulations...maybe a different word is priorities in the future for research funding, policy is a tricky word.

Federal staff: There were disagreements about how much to include in the document on policy concerns and recommendations, were they appropriate to include at all, would they jeopardize acceptance of the report by the government, you know? Was the mandate, the charter that we were given, the language in the legislation, such that it was appropriate to even include those topics, some people said no, and others said, well listen, if you’re going to talk about research gaps, research needs, you know you can’t avoid talking about policy issues.

Advocate: I mean it went from “we can’t talk about prevention” to “the report is named *Prioritizing Prevention.*” So I felt really good about that, there were people who didn’t think that policy had any place in it at all … that this prestigious committee of experts shouldn’t touch policy, that we shouldn’t talk about the implications for policy.

A final issue pertinent to the challenge of implementation is the lack of adequate, accessible information on government spending and activities pertinent to breast cancer research. Almost all IBCERCC members noted that it was difficult to know what changes had taken place within government as a result of the report. Some federal IBCERCC members noted that they only knew of certain impacts because they were inside government institutions. One interviewee said it would be almost impossible for non-governmental employees to have access to the information on funding shifts in response to reports such as the IBCERCC that federal employees had. This dynamic compounds the IBCERCC committee’s struggle to ascertain the actual dimensions of federal funding for breast cancer prevention pertinent to environmental contributors (and specifically what funding was allocated to voluntary exposure vs. non-voluntary exposure), illustrating an ongoing lack of searchable, suitably coded data on public cancer research.

## Discussion

Qualitative social science offers insight into the politics of scientific evidence, insights that can aid environmental and public health experts in navigating processes of scientific consensus building [28–30]. Generally, how one defines a problem or selects a “diagnostic frame,” informs how one approaches a solution or a “prognostic frame” [31]. Interviews revealed two primary sets of conflicting diagnostic and prognostic frames. Echoing Brown’s dominant epidemiological paradigm and public paradigm distinctions, one group tended to seek incremental changes to research, funding, and spoke little of policy, while a second group called for more profound changes in research paradigms and a precautionary approach to chemical regulation. Academic scientists, agency staff, and advocates adhered to *distinct conceptions* of the following: the report mandate and appropriate scope, how to interpret different types of scientific evidence on environmental breast cancer causes, whether and how science should inform policy, and implicitly, how changes in policy, advocacy, and research funding come about. For some, the IBCERCC report can serve as a tool for advocates to educate consumers on purchasing choices or as evidence to be used to “hold the government’s feet to the fire,” and compel more health-protective regulatory policy. For others, the science is in its infancy, and scientists must continue to look for stronger evidence, a “smoking gun,” entailing research over longer time periods and during critical windows of exposure. At the federal agency level, some felt that each agency could only do its own part, and that depended significantly on the current leadership of the agency. A smaller number of interviewees called for significant reform of TSCA, the EPA, and one scientist called for “stopping the flow of this fire hydrant of carcinogenic chemicals released into the economy in the first place.”

The concept of “hidden arguments” assists in understanding the distinct opinions expressed by interviewees regarding how research, policy, funding, and advocacy intersect and influence one another [25]. Tesh writes that hidden arguments are “political ideology about what constitutes legitimate sources of knowledge … these hidden arguments, or conceptual frameworks, inform which questions get asked, which do not, and how researchers go about investigating them. Furthermore, these frameworks theorize about the causes of disease, so they influence how researchers conceptualize and operationalize the determinants of health.” [25]. In assessing the impact of the IBCERCC report, Tesh’s discussion of hidden arguments serves as a framework for understanding the tensions between the environmental breast cancer movement and adherents of more dominant narratives of epistemological objectivity and institutional change as incrementally building on extant scientific knowledge of environmental risks. For Brown, dominant pluralist paradigms can be deployed to stifle more substantive actions capable of addressing political economic drivers of environmental chemical contamination [5, 25]. Regarding concerns over non-voluntary chemical exposure, using the diagnostic frame of preventing breast cancer as a sole matter of “innovative science” could delay action by absolving stakeholders such as industry and federal regulators of responsibility for addressing immediate issues like substantive TSCA reform (and health-protective implementation), let alone the current political-economic trends toward weakening federal regulatory policy and research capacity. If a lack of science is the diagnostic or problem frame, the prognostic frame or solution is doing more science. Such framing elides politics and perhaps most importantly private sector agendas and interests inside federal regulatory agencies in the U.S.

As discussed above, almost all the academic and federal interviewees discussed the impact of government budget crises on hindering the success of implementing the recommendations of the IBCERCC. For the advocates, select academics and federal interviewees, social and policy change are generally understood to be power struggles. These interviewees were aware of the history of the breast cancer movement(s) that used non-institutional direct action, non-violent protest, and institutional (lobbying, public testimony, etc.) tactics to secure federal research funding. Thus, they perceived the importance of taking active and decisive responsibility for shaping the current state of scientific knowledge on breast cancer. These actors foresaw significant work ahead to make progress, invoking a conflict or power-based model of change, which the health social movements literature largely supports [5, 9, 10, 32, 33].

Some scientists and federal stakeholders interviewed adhered to pluralist models of social and political change, conceptualizing of science as speaking for itself. Here individuals pursue scientific research that may eventually be taken-up by policy makers and regulators [5, 34]. These interviewees appeared more resigned to budget constraints in the near future, in contrast to advocates who were more likely to focus on specific policies, reforming the EPA, or industry interests during interviews. While there were some exceptions, federal members and scientists conceptualized of institutional change from a more pluralist perspective, not drawing attention to industry behavior in a) producing growing volumes of carcinogens and endocrine disrupting chemicals or b) erecting barriers to adequate regulation, oversight, or monitoring of known harmful compounds. Many interviewees across all stakeholder groups discussed getting individuals in the general public information in order to better protect themselves, using consumer movements to shift policy towards protection, and voicing an “all you can do is your part” sentiment on multiple scales. One advocate expressed concern that younger generations were not willing to sacrifice and fight politically the way her generation had on breast cancer in the early 1980s. She felt resigned to the breast cancer movement shifting into a less radical, consumer-oriented movement among mothers concerned with purchasing safe products. This concern over the decline of a more politically engaged breast cancer movement was recently echoed by a scientist lamenting the closure of the BCERP grant program and lack of broad outcry in 2019. Thus, interviewees adhered to fundamentally conflicting conceptions of social, scientific, and political change.

## Conclusion

The short and long-term impacts of the IBCERCC report are mixed, with stakeholder assessments varying widely. In the challenging funding climate for federal research in 2014, committee members saw the continuation of the BCERP and a companion research center as important short-term outcomes of the IBCERCC. According to one federal interviewee, it is unusual for a Federal Advisory Committee Act (FACA) to function across federal agencies and bring agency staff together with scientists and advocates to share their work, tackle questions regarding funding allocation, and produce a collaborative set of future recommendations. However, as numerous interviewees noted, the power of this model and collaborative effort risk being lost without accountability, especially to public health oriented stakeholders. Just as interviewees had different understandings of barriers to the implementation of report recommendations, interviewee comments varied in their implicit models of change. To some committee members, it was not clear to them who or what entities were responsible for carrying out the recommendations. However, many advocates, select federal staff and scientists felt that they were responsible for “carrying this on their backs” to ensure it had impacts on research, policy, and funding. Looking at the impetus behind what ultimately became the IBCERCC, this assessment appears to be historically accurate. For some scientists, with the publication of the 2013 report, they considered their work on the committee done; they had assessed research funding and the scientific literature, stating that it is up to advocates, policy makers, and regulators to decide what to do with the science. The NBCC meanwhile has moved into advocating and supporting research they believe will produce the quickest and most effective breast cancer solutions, including preventative vaccines. NBCC’s current focus is on finding medical solutions to breast cancer by 2020 [22], in contrast to the focus on chemical exposures held by most other advocates interviewed.

These stakeholder interviews reveal significant contrasts regarding how IBCERCC members, breast cancer researchers, advocates, and funders conceive of their roles in bringing about necessary changes to address breast cancer prevention. Most IBCERCC participants believed that the committee’s mandate was worthwhile, though some believed that their goals had a slim chance of implementation, with one committee member guessing that only 10% of these types of reports had any impact. This assessment indicates that the IBCERCC, PCP, and IOM reports represent greater acknowledgment of the significance of environmental contributors to breast cancer by the federal government and large research establishments, long called for by groups within breast cancer movement(s). However, short and longer-term outcomes indicate that social movement advocates will need to continue to play a central role in driving institutional responses to the global prevalence of breast cancer. Integrating social science into multi-stakeholder scientific collaborations offers opportunities for examining underlying assumptions, implicit theories of change, and the identification how assumptions impact problem definitions, the scope of solutions considered, and the necessity of systems of accountability.

## Additional file

Additional file 1: Interview Questions for IBCERCC Research Project. (DOCX 60 kb)

## Abbreviations

BCERP: Breast Cancer and the Environment Research Program
CDC: Centers for Disease Control
DEP: Dominant Epidemiological Paradigm
DOD: Department of Defense
EPA: Environmental Protection Agency
FACA: Federal Advisory Committee Act
IBCERCC: Interagency Breast Cancer and Environment Research Coordinating Committee
IOM: Institutes of Medicine
NBCC: National Breast Cancer Coalition
NCI: National Cancer Institute
NGO: Non-governmental Organization
NIEHS: National Institute of Environmental Health Sciences
NIH: National Institutes of Health
NTP: National Toxicology Program
PCP: President’s Cancer Panel
TSCA: Toxic Substances Control Act
