# Supplementary material for: Challenging dominant breast cancer research agendas: perspectives on the outcomes of the interagency breast cancer and environment research coordinating committee
Source: Environ Health. 2019 May 6;18:41. doi: 10.1186/s12940-019-0479-1 (PMC6501325; doi:10.1186/s12940-019-0479-1)
Supplement: Supplementary file 1 — Interview Questions for IBCERCC Research Project. (DOCX 60 kb) [file 12940_2019_479_MOESM1_ESM.docx]

**Semi-Structured Interview Questions for IBCERCC Research Project**

1. How did you first get involved in the Interagency Breast Cancer and Environment Research Coordinating Committee (IBCERCC)?

[For non-IBCERCC committee members, what is the nature of their awareness of IBCERCC or involvement with the 2010 President’s Cancer Panel or 2012 Institute of Medicine environment and cancer initiatives].

1. Please tell me in your own words what the report found.
2. What were your initial expectations?
3. [IBCERCC Committee Members] When you were first asked to be on the committee, what did you think it could or should do? Did this change once your joined the committee?
4. When you began, what impact did you think you would have, and what impact did you actually have?
5. [IBCERCC Committee Members] What kinds of issues came up in the deliberations? Was there disagreement?
6. [IBCERCC Committee Members] Did any organizations or agencies have more power or influence than others?
7. How did you feel about the final report?
8. [IBCERCC Committee Members] How has your participation on the IBCERCC affected your organization?
9. How effective was the dissemination of the report?
10. Did the report get adequate attention?
11. How will the report impact policy? Funding? Advocacy?

*If individual answers “not significantly” to question #12, follow-up with:*

1. What would have increased the report’s impact?
2. What could the committee have done differently to facilitate impacts on policy, funding, and advocacy?
3. Will there be any follow-up activities?
4. Can you describe what you individually or your organization has done to implement recommendations found in the report?
5. Is there anything we have not covered that you would like to add?
